# Patient safety considerations concerning the scheduling of emergency‐off system tests

**DOI:** 10.1120/jacmp.v15i2.4583

**Published:** 2014-03-06

**Authors:** Ivan A. Brezovich, Richard A. Popple

**Affiliations:** ^1^ Department of Radiation Oncology The University of Alabama at Birmingham Birmingham AL USA

**Keywords:** emergency‐off system, emergency‐stop system, patient safety, quality assurance

## Abstract

Emergency‐off systems (EOS) are essential to the safe operation of medical accelerators and other high‐risk equipment. To assure reliable functioning, some states require weekly tests; others permit monthly, tri‐monthly or even six‐monthly tests, while some do not specify test intervals. We investigate the relative safety of the various test schedules by computing the fraction of time during which a nonfunctional state of the EOS may remain undetected. Special attention is given to the effect of flexibility (i.e., to regulations that specify the number of tests that have to be done in any given time interval, but allow a range within the interval during which a test can be done). Compared to strict test intervals, a schedule that provides flexibility increases risk only marginally. Performing tests on any arbitrary day of the week when weekly tests are required increases the time span during which a nonfunctionality goes undetected by only 17%, compared to an exact one‐week schedule. The same ratio applies for monthly tests. For a three‐month schedule, the relative risk increases by only 2% if tests are done on an arbitrarily chosen day during each due‐month, compared to tests done on an exact three‐month schedule. The most irregular time intervals possible in a three‐calendar month schedule increase the relative risk by 11%. For the six‐month and the 12‐month schedule the ratio of risks is even smaller. The relative risk is virtually independent of the mean time between failures of the EOS, but the absolute risk decreases in proportion the mean time between failures. Adherence to strict, resource‐intensive test intervals provides little extra safety compared to flexible intervals that require the same number of tests per year. Regulations should be changed to provide the practicality offered by flexible test schedules. Any additional increase in patient safety could be achieved by strict regulations concerning reliability of emergency‐stop (e‐stop) systems.

PACS numbers: 87.55.N‐, 87.55.Qr, 87.56.bd

## I. INTRODUCTION

Emergency‐off systems (EOS) are provided on medical accelerators and simulators so that the operator can stop the production of X‐rays and mechanical movements if the normal controls malfunction. Furthermore, to protect against electric shock hazards, an activated EOS removes electricity from the machine by tripping the main circuit breaker. To assure functionality, many states require EOS tests at specified intervals, ranging from once a week,^(1)^ once a month,^2^, ^3^, ^4^ every three months,^5^, ^6^, ^7^, ^8^, ^9^, ^10^ to every six months.^11^, ^12^ Colorado regulations^(13)^ refer to TG 40,^(14)^ while giving the physicist some discretion. California requires a Radiation Safety and Protection Program, but is nonspecific concerning the EOS. Missouri regulations^(15)^ do not mention the EOS.

The wide range of test schedules may have been influenced by the AAPM and CRCPD (Conference of Radiation Control Program Directors) recommendations effective at the time when the State regulations were passed. The AAPM report 13 of 1984 suggests weekly tests;^(16)^ TG 40 recommends monthly tests;^(14)^ TG 142^(17)^ accepts manufacturers' recommendations, while the CRCPD suggests three‐month intervals.^(18)^

While one may argue about the merits of frequent EOS tests, the requirement for strict test intervals puts substantial strain on personnel and resources compared to a flexible schedule which would allow tests to be done within a reasonable time span. One time our department, which has to follow a three‐month schedule, was cited for exceeding the due‐date by one day. We felt that such a strong reprimand was not justified by the very small increase in risk of a one‐day delay. In this paper, we analyze the likelihood of EOS failures, with special emphasis on flexible test schedules.

## II. METHODS

### A. Effect of EoS tests

When an EOS is activated on a modern accelerator, all systems are shut down and it takes typically 15 minutes to bring the machine back to clinical service. Furthermore, the sudden removal and reapplication of power associated with an EOS test puts substantial stress on an accelerator. To assure uninterrupted patient care, EOSs are preferably tested by a service engineer who can make the necessary repairs if an accelerator is damaged by a test.

If the service engineer is unavailable at the due date, the institution is faced with either being in violation of regulations or delaying patient treatment, which has the potential to compromise tumor control.^(19)^ Providing a cushion by doing the tests a few days ahead of the due date avoids this problem, but causes creep toward an earlier week or month, disrupting the normal rhythm. This predicament could be avoided by regulations permitting a range of test dates (e.g., requiring tests to be done every third calendar month rather than exactly every three months).

The drawback of a flexible schedule is that some intervals between consecutive tests can be appreciably longer than the intervals provided by a strict schedule. For example, if in a three‐calendar‐month schedule a test is done on July 1st and the subsequent test in October is delayed till the end of the month, a total of 119 days will have passed between the two tests, increasing the risk of an undetected EOS failure. (For simplicity, we neglect the unequal lengths of months and assume all to have 30 days.) To get back to the normal schedule, the next test may be done at the beginning of January, so that only 61 days will have passed between tests, leading to a lower risk of an undetected failure during the shortened interval. Thus, the increased risk during longer intervals is partially offset by a lower risk during shorter intervals.

### B. Downtime of emergency‐off systems

The probability that an EOS will be operational t days after a successful test is given by

(1) $$P_{op}^{EOS}=e^{-\lambda t}$$

where

(2) $$\lambda =\frac{1}{MTBF_{EOS}}$$

and $MTBF_{EOS}$ is the mean time between failures of the EOS. The mean time between failures of any component or system, MTBF, is defined in engineering as the average, or expected value, of operating times between failures of a repairable item.^(20)^

The probability that the system will not be working t days after the test is then 1 minus the probability that it will be working. By integrating that probability with respect to time, we get the expected inoperative time within the time span between a given test and the next test N days later:

(3) $$T_{inop}(N)=\int_{0}^{N}(1-e^{-\lambda t})dt$$

Solution of Eq. (3) yields

(4) $$T_{inop}(N)=N-\frac{1}{\lambda }(1-e^{-\lambda N})$$

Since the test interval *N* is typically much shorter than the mean time between failures of the EOS,

(5) λN≪1

Eq. (4) can be expanded in a Taylor series and only the dominant terms kept. With this approximation,

(6) $$T_{inop}(N)=\frac{1}{2}\lambda N^{2}$$

**Figure 1:**
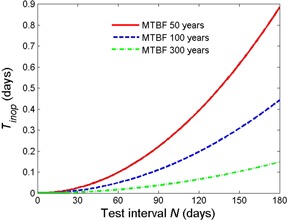
Expected length of time during which an EOS is nonfunctional following a successful test.

A plot of the downtime computed from Eq. (4) is depicted in Fig. 1. It shows that the expected time span during which an EOS failure remains undetected increases rapidly with the time that passed after a successful EOS test. For a mean time between failures of one hundred years, for example, we can expect the EOS to be nonfunctional for an average of about 0.1 days during a 90‐day interval following a successful test, and about twice as long if the interval is increased to 120 days.

**Figure 2:**
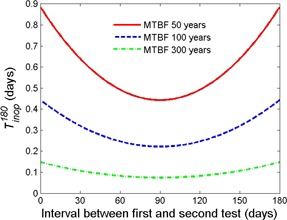
Expected downtime of an EOS during a 180‐day period for a three‐month test schedule with a variable test interval. The initial and final tests occur as scheduled at days 0 and 180, respectively, while the intervening test occurs at a variable time.

The effect of unequal test intervals is illustrated in Fig. 2 for a three‐calendar‐month schedule. Tests are scheduled on the first day of the respective due month. We assume that the first test is done as planned, the second is delayed, while the third test is again done as scheduled on day 181. If the second test had not been delayed, 90 days would have passed since the first one, and the inoperational time of the EOS system would have been least. However, assuming that the second test was delayed to the end of the due month, to day 120, a total of $N_{1}=119$ days have passed since the first test. Compared to the strict three‐month schedule, the delay permitted by the three‐calendar‐month schedule increased undetected inoperational time by about 11%.

**Figure 3:**
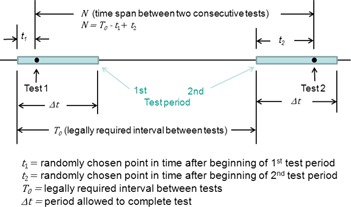
Schematic of flexible EOS test schedule.

If permitted by flexible regulations, EOS tests would likely be done on random days during each due period, as shown in Fig. 3. Let $T_{0}$ be the required time interval between tests (e.g., three months in a quarterly schedule) and Δt the period allowed to complete the test (e.g., one month in a quarterly schedule). For EOS tests conducted at time $t_{1}$ after the beginning of a test period and at $t_{2}$ after the beginning of the subsequent period, the time interval N between the two tests is

(7) $$N=T_{0}-t_{1}+t_{2}$$

For tests occurring with equal probability within the period Δt, the mean time the EOS is not operational is given by integrating Eq. (4) as

(8) $$T_{inop}=\frac{1}{\Delta t^{2}}\int_{0}^{\Delta t}\int_{0}^{\Delta t}[N+\frac{1}{\lambda }(e^{-\lambda N}-1)]dt_{1}dt_{2}$$

The double integral in Eq. (8) can be readily carried out and yields

(9) $$T_{inop}=T_{0}-\frac{1}{\lambda }-\frac{2}{\lambda^{3}\Delta t^{2}}e^{-\lambda T}[1-\text{cosh}(\lambda \Delta t)]$$

Using a Taylor expansion with the condition given by Eq. (5) and keeping only the dominant terms simplifies Eq. (9) to

(10) $$T_{inop}=\frac{\lambda }{2}(T_{0}^{2}+\frac{1}{6}\Delta t^{2})$$

Equation (10) shows that a flexible schedule adds only insignificantly to an undetected down‐time of the EOS, compared to the strict schedule (Δt=0). Intuitively this can be understood by the much larger number of combinations of test days that yield relatively even spacing between tests compared to combinations that yield extremely long and short intervals.

Dividing both sides of (9), (10) by the test interval $T_{0}$ yields $F_{\text{inop}}$, the fraction of time during which an EOS is nonfunctional:

(11) $$\frac{T_{inop}}{T_{0}}F_{inop}=\frac{\lambda }{2}(T_{0}+\frac{1}{6}\frac{\Delta t^{2}}{T_{0}})$$

Equation (11) indicates that risk is proportional to the length of the test interval. $F_{\text{inop}}$ is also the probability that the EOS is inoperational at any given point in time.

A comparison of risks of various test schedules, expressed by the number of days per year during which a failed EOS may go undetected, is presented in Table 1. For the weekly tests, the regular (strict) schedule consists of a test on the same day of each week. The flexible schedule would require one test per week on any desired day of the week. The most irregular (worst) interval permitted by a “calendar week” schedule consists of two tests on consecutive days, followed by a 13‐day period to the next test. Similar considerations apply to the monthly schedule. For the three‐, six‐, and 12‐month schedules, it is assumed that the test is done either on the same day of the due month (strict), at random days within the due month (flexible), or at days within consecutive due months that yield the largest variations in the intervals between tests (worst).

**Table 1:** Average number of days per year during which the nonfunctionality of an EOS remains undetected

		*EOS Mean Time Between Failures (years)*							
*Schedule* ^a^		*1*	*5*	*10*	*20*	*50*	*100*	*1000*	*10000*
Weekly	Strict	3.49	0.70	0.35	0.18	0.070	0.035	0.004	$<10^{-3}$
	Flexible	4.06	0.82	0.41	0.20	0.082	0.041	0.004	$<10^{-3}$
	Worst	6.93	1.40	0.70	0.35	0.14	0.070	0.007	$<10^{-3}$
Monthly	Strict	14.8	3.03	1.52	0.76	0.30	0.15	0.015	0.002
	Flexible	17.1	3.53	1.77	0.89	0.35	0.18	0.018	0.002
	Worst	28.8	6.02	3.03	1.52	0.61	0.30	0.030	0.003
Every 3 months	Strict	42.1	8.98	4.53	2.27	0.91	0.46	0.046	0.005
	Flexible	42.7	9.14	4.61	2.32	0.93	0.46	0.046	0.005
	Worst	46.0	9.95	5.02	2.52	1.01	0.51	0.051	0.005
Every 6 months	Strict	77.8	17.7	8.98	4.53	1.82	0.91	0.091	0.009
	Flexible	78.1	17.7	9.02	4.55	1.83	0.92	0.092	0.009
	Worst	79.4	18.1	9.22	4.65	1.87	0.94	0.094	0.009
Every 12 months	Strict	134	34.2	17.7	8.98	3.63	1.82	0.18	0.018
	Flexible	134	34.2	17.7	8.99	3.63	1.82	0.18	0.018
	Worst	135	34.4	17.8	9.04	3.65	1.83	0.18	0.018

^a^ The strict schedule is for exact intervals (Eq. 6), the flexible schedule is for tests occurring randomly within test periods at the specified interval (Eq. 11), and the worst‐case schedule is for tests that alternate between the minimum and maximum intervals of the flexible schedule. The flexible test period is one week for the weekly schedule and one month for the remaining schedules.

### C. Effect on patient safety

An EOS failure can put a patient at risk only if the treatment delivery system that normally terminates the production of X‐rays and mechanical motions fails during the short fraction of time when the EOS is nonfunctional. Thus, the mean time between such incidents, MTBI, is given by

(12) $$MTBI=MTBF_{treatment}\times \frac{1}{F_{inop}}$$

where ${\text{MTBF}}_{\text{treatment}}$ is the mean time between failures of the treatment delivery system. Table 2 shows expected MTBI for various test schedules and MTBF of the EOS. In each case, the ${\text{MTBF}}_{\text{treatment}}$ was taken as 100 years. According to Table 2, an EOS having a MTBF of 100 years would lead to an incident about every 20,000 years if a 12‐month test schedule were used. A three‐month schedule would reduce the number of incidents to one every 78,000 years, whereas weekly tests could achieve one‐million machine years without incident. Note that there is virtually no difference in failure rates between strict and flexible test schedules for test intervals of three months and longer. Only the most irregular schedule decreases the respective MTBI by slightly less than a factor of 2 when weekly or monthly schedules are used.

**Table 2:** Mean time between patient endangerment incidents (MTBI) in thousands of years as a result of treatment system failure when the EOS is not functional. The mean time between failures of the treatment system is assumed to be 100 years

		*Mean Time Between EOS Failures (years)*							
*Schedule* ^a^		*1*	*5*	*10*	*20*	*50*	*100*	*1000*	*10000*
Weekly	Strict	10	52	104	208	520	1040	10400	10^6^
	Flexible	9.0	45	89	178	446	892	8914	89151
	Worst	5.3	26	52	104	260	520	5200	52000
Monthly	Strict	2.5	12	24	48	120	240	2400	24000
	Flexible	2.1	10	21	41	103	206	2057	20572
	Worst	1.3	6.1	12	24	60	120	1200	12000
Every 3 months	Strict	0.9	4.1	8.1	16	40	80	800	8000
	Flexible	0.9	4.0	7.9	16	39	79	786	7855
	Worst	0.8	3.7	7.3	14	36	72	720	7200
Every 6 months	Strict	0.5	2.1	4.1	8.1	20	40	400	4000
	Flexible	0.5	2.1	4.0	8.0	20	40	398	3982
	Worst	0.5	2.0	4.0	7.9	20	39	389	3892
Every 12 monthd	Strict	0.3	1.1	2.1	4.1	10	20	200	2000
	Flexible	0.3	1.1	2.1	4.1	10	20	200	1998
	Worst	0.3	1.1	2.1	4.0	10	20	199	1986

^a^ The strict schedule is for exact intervals (Eq. 6), the flexible schedule is for tests occurring randomly within test periods at the specified interval (Eq. 11), and the worst‐case schedule is for tests that alternate between the minimum and maximum intervals of the flexible schedule. The flexible test period is one week for the weekly schedule and one month for the remaining schedules.

## III. DISCUSSION

In the context of this article, the term MTBF has to be interpreted as the likelihood that one system in a group of many will fail during any given year, not as the expected lifetime of an individual system. Aging of materials, as well as wear and tear due to usage, keep lowering the MTBF and increasing the stochastic probability of failure as the system gets older. Table 2 provides data for a wide range of MTBFs.

An accurate computation of safety would require knowledge of the MTBF for the accelerator and the EOS. For some simple devices such information is supplied by the manufacturer. Ball bearings, for example, are characterized by the load that 90% of bearings of a large sample could withstand for one million revolutions. Mathematical expressions are provided for computing the “expected life” at the actual (usually much lower) load, speed (revolutions per minute), and operating temperature. During the expected life the failure rate is purely statistical, we only know that 90% of bearings will survive, but an individual bearing can fail at any time. Due to wear, fewer than 90% of the remaining bearings would survive another equal time of operation (i.e., the failure rate increases with time in service and the MTBF decreases).

To make a similar risk analysis for accelerators, one would have to know the probability that any relay, switch or other vital component in the system EOS will fail as a function of chronological age, number of switching cycles, and environmental conditions. Combined with information about the probability that the normal system fails, one could use Table 2 to compute the likelihood that a patient would be endangered by an EOS malfunction. As the reliability and the MTBF of the EOS decreases due to age and use, Table 2 provides a guideline to determine the increase in the number of tests required to maintain the desired level of safety. Alternatively, one could replace aging components to restore the MTBF of the EOS to its original level.

Unfortunately, such detailed knowledge is not available for complex systems like accelerators. Considering that there are about 10,000 accelerators in use worldwide and assuming a MTBF of 100 years for the treatment delivery system and the EOS and a three‐month EOS test schedule, one total failure would occur every eight years. Since there is no evidence for such a high rate of total failures, one can assume that MTBFs of the normal delivery system and/or the EOS are longer than 100 years.

To obtain an evidence‐based estimate of the actual failure rate of EOS, we followed the recommendation of one of the referees and posted an online survey to the medical physics list server.

The survey asked the following questions:

1. How many linac‐years of experience do you have (linacs in clinic × years in clinic)?
2. How many times has an emergency‐off system failed during a routine test?
3. How many times has the emergency‐off system been used successfully to protect a patient?
4. How many times has the emergency‐off system been needed to protect a patient but failed?

Respondents had the options of including comments and providing contact information. A summary of the responses is shown in Table 3.

Ninety‐seven survey results were received, totaling 4110 linac‐years. One respondent reported 600 linac‐years of experience and zero events for questions 2‐4. The respondent did not provide contact information and so we were unable to confirm that this number was correct. Because 600 linac‐years comprised about 15% of the total experience in the survey, we censored this response and provide data including and excluding this response. Excluding this response, the median experience was 24 linac‐years. There was only one reported instance when the EOS was needed to protect a patient and failed, resulting in death. The fatality happened in the 1970s on a cobalt unit when a motion relay welded. The machine turned off with e‐stop, but motion started again with e‐stop being released, crushing the patient's chest.

Our institution has not experienced a failure during more than 130 machine‐years. However, part of the button of a wall switch in a ${\;}^{60}\text{Co}$ room did break off during a test many years ago, probably due to age and deterioration of the radiation sensitive plastic by 15 years of exposure to scattered radiation. In an emergency situation, it may have been difficult to activate that switch. A service engineer (Mike Williams, private communication, 2010), responsible for maintaining more than ten accelerators, experienced one EOS failure during a routine test during his 30+ year career, but in the “safe” mode. Resetting the wall switch after a test did not bring the accelerator back to operation. He, too, attributed the failure to deteriorated plastic parts within the switch. Such information implies that normal and e‐stop systems on currently used accelerators fail less frequently than once every 100 accelerator‐years.

To further enhance safety, EOSs of modern accelerators incorporate redundant components. They are typically equipped with a DC power supply located near the operator console. The DC power has to pass through all EOS switches before reaching the main circuit breaker on the wall and the accelerator. A malfunction of the DC supply or activation of an emergency‐off switch trips the main circuit breaker, removing all electrical power going to the accelerator. In addition, numerous relays and systems within the accelerator that provide power and control for mechanical motion and beam generation are de‐energized. The large distance between the DC power supply and the accelerator makes it virtually impossible for a short circuit or other component failure to continue providing DC power to the main circuit breaker and the accelerator after the EOS has been activated.

**Table 3:** Survey results of accelerator e‐stop failures

	*Linac‐years*	
	*4110*	*3510*
Number of EOS test failures	21	21
Number of EOS patient protection	33	33
Number of patient endangerments due to EOS failure	1	1
Mean time between EOS failures (years)	186.8	159.5
Mean time between treatment system failures (years)	120.9	103.2
Mean time between patient endangerments (years)	4110.0	3510.0

To bring an accelerator back to operation, the emergency‐off switch has to be reset by the operator (not just released), the main circuit breaker on the wall has to be reset, and the internal mechanism of the accelerator has to be restarted. Considering these new safety features, an accident similar to the one on the cobalt unit is most unlikely.

In view of the low failure rates and the small gain in safety offered by rigid test schedules, the extra burden imposed by strict schedules compared to flexible test intervals appears unjustiacm20327fied. Concerning frequency of tests, weekly tests seem safest. However, the wear on the EOS imposed by each test reduces the MTBF for the remainder of the service life, so that the weekly schedule may eventually become less safe than one involving fewer tests. Furthermore, the performance of components of the accelerator that are not part of the EOS may be compromised by the stress of repeated removal and reapplication of power, outweighing any potential benefit of unnecessarily frequent testing.

Potential weak points in the current system are nonuniform standards for the wall‐mounted emergency‐off switches, which constitute single points‐of‐failure. These switches are usually supplied by builders of the accelerator vaults who may not be familiar with the harsh radiotherapy environment.

Rather than requiring very frequent EOS tests, it seems that safety and practicality would be better served by ensuring that EOSs, especially the wall‐mounted switches, fail in the “safe” mode. Many State regulations use wording like “all safety interlocks shall be designed so that any defect or component failure in the safety interlock system prevents or terminates operation of the accelerator.”^21^, ^22^, ^23^, ^24^, ^25^, ^26^ In fact, if safety could be legislated (i.e., if manufacturers were able to fully comply with the regulations and make EOS systems that absolutely never fail in the unsafe mode), EOS tests would be unnecessary in those states.

While perfect safety can never be achieved, long‐term reliability of switches could be enhanced by use of radiation‐resistant materials with proven longevity. To assure failure in a safe mode, contacts could be spring‐loaded toward the open position and kept closed by an internal mechanism. Deterioration of the switch would cause the springs to pull the contacts apart and thereby interrupt DC power and shut down the accelerator.

## V. CONCLUSIONS

We have shown that rigid test intervals for emergency‐off systems provide negligible gain in safety compared to flexible schedules that specify the over‐all frequency of tests, but allow time intervals for doing the individual tests. This applies to all test schedules, including weekly, monthly and tri‐monthly. Until State regulations catch up with today's technology, radiation safety inspectors can contribute to quality patient care by showing lenience in enforcing outdated rigid test schedules. Regulators should also reconsider the requirement for weekly, monthly or even tri‐monthly tests, since there is no evidence that such short test intervals provide more patient safety than longer ones.

## References

[1] New York . NY City Health Code. Article 175 – Radiation Control. §175.64(g)(9)(vi) and §175.64(g)(9)(vii)(F). 6577360

[2] Florida Administrative Code . Florida Department of Health. Control of Radiation Hazards: Operating procedures. Chapter 64E–5.808 (3).

[3] State of Georgia . Radiation Safety Requirements for Particle Accelerators. Rules and Regulations, 290‐5‐22.05(9)(c).

[4] Illinois Administrative Code . Emergency Management Agency. Title 32, Chapter II, Subchapter b, Part 360.120 g) 1) D).

[5] Alabama Administrative Code . Radiation Control, Chapter 420‐3‐26‐.09‐(7)(a).

[6] Mississippi Department of Health . Title 15, Part 21, Subpart 78, Chapter 1, Code Ann. §45‐14‐11 Rule 1.9.10.3.

[7] Administrative Rules of Montana . Radiation Control, Rule 37.14.1426(3).

[8] Oregon Administrative Rule 333‐109‐0015(6).

[9] State of Rhode Island . Rules and regulations for the control of Radiation, R23‐1.3‐RAD Part D.36(c).

[10] Texas Administrative Code 25, §289.229(f)(3)(A)(viii).

[11] The North Carolina Radiation Protection Commission , 15A NCAC 11.0908 (c).

[12] State of South Carolina . Rules and Regulations for Radiation Control, Title C, Part III, Radiation Safety Requirements for Particle Accelerators, RHC 3.4.8.

[13] Colorado Department of Public Health and Environment . Radiation Control, Part 24 – Particle Accelerators and Therapeutic Radiation Machines in the Healing Arts, Periodic Quality Assurance Checks 24.8.20.7.

[14] Kutcher GJ , Coia L , Gillin M , et al. Comprehensive QA for radiation oncology: Report of AAPM Radiation Therapy Committee Task Group 40. Med Phys. 1994;21(4):581–618. 8058027 10.1118/1.597316

[15] Missouri Code of State Regulations . Chapter 10, Title 19 CSR 20‐10.180: Requirements for radiation therapy installations.

[16] Radiation Therapy Committee Task Group #24. Physical aspects of quality assurance in radiation therapy. AAPM Report 13. Madison (WI): Medical Physics Publishing; 1984.

[17] Klein EE , Hanley J , Bayouth J , Yin FF , et al. Task Group 142 report: Quality assurance of medical accelerators. Med Phys. 2009;36(9):4197–212. 19810494 10.1118/1.3190392

[18] Conference of Radiation Control Program Directors , Suggested State Regulations for Control of Radiation, Part I, Sec. I.10 c, 1991 Available from: http://www.crcpd.org/SSRCRs/ipart91.PDF

[19] Bese NS , Hendry J , Jeremic B . Effects of prolongation of overall treatment time due to unplanned interruptions during radiotherapy of different tumor sites and practical methods for compensation. Int J Radiat Oncol Biol Phys. 2007;68(3):654–61. 17467926 10.1016/j.ijrobp.2007.03.010

[20] ANSI/IEEE . IEEE Guide for general principles of reliability analysis of nuclear power generating station safety systems. ANSI/IEEE Std 352‐1987. Piscataway, NJ: IEEE; 1987 Available from: http://ieeexplore.ieee.org/stamp/stamp.jsp?tp=&arnumber=159367

[21] New York. NY City Heatlh Code, Article 175 Radiation Control, §175.64(g)(5)(i)(I). 6577360

[22] Mississippi Department of Health . Title 15, Part 21, Subpart 78, Chapter 1, Code Ann. §45‐14‐11 Rule 1.9.8.4.

[23] Administrative Rules of Montana . Radiation Control, Rule 37.14.1418(5).

[24] Oregon Administrative Rule 333‐109‐0030(4).

[25] State of Rhode Island . Rules and regulations for the control of Radiation, R23‐1.3‐RAD Part D.34(e).

[26] The North Carolina Radiation Protection Commission . 15A NCAC 11.0906 (e).

